# Selected Trace Metals in Four Major Surface-Water Reservoirs of the Houston Metropolitan Area

**DOI:** 10.1007/s00128-026-04358-0

**Published:** 2026-10-30

**Authors:** Ali D. Alshehri, Sonya Good, Jacob Wei, Tuan Phan

**Affiliations:** Department of Chemistry, Texas Southern University, Houston, TX USA

**Keywords:** Trace metals, Surface water, ICP-MS, Houston reservoirs, Water quality

## Abstract

This technical note reports selected trace metals in four major surface-water reservoirs associated with the Houston metropolitan region. Shoreline surface-water samples from Lake Houston, Lake Livingston, Lake Conroe, and Clear Lake were analyzed for As, Cd, Cu, Pb, Ni, and Zn by inductively coupled plasma-mass spectrometry (ICP-MS) following U.S. EPA Method 200.8 quality-control procedures. Copper was the dominant analyte in all reservoirs, with mean concentrations of 9.82–15.5 µg L⁻¹. Measured concentrations were low, and arsenic and lead were below relevant federal drinking-water benchmarks. These results provide a concise descriptive baseline for future reservoir monitoring.

## Introduction

Urban reservoirs receive trace metal inputs from natural geologic sources, atmospheric deposition, roadway runoff, shoreline development, wastewater infrastructure, marina activity, and industrial land use. Although concentrations may remain below regulatory benchmarks, reservoir-specific metal patterns can provide screening-level information for identifying analytes that warrant continued monitoring. Such baseline data are useful in large metropolitan watersheds where industrial, transportation, municipal, and recreational activities overlap with public water-supply and coastal-maritime settings (Tchounwou et al. 2012; Houston Public Works 2024; TCEQ 2024; Das et al. 2026). Previous investigations have documented water-quality conditions in Houston-area reservoirs. Van Metre and Sneck-Fahrer (2002) examined historical contaminant trends in Lake Houston sediments, while Adams (2025) evaluated spatial and seasonal water-quality patterns, including selected trace metals, in Lake Conroe. These studies provide useful regional context, although differences in sampling media, locations, and analytical approaches limit direct comparisons with the present surface-water measurements.

The Houston metropolitan region includes major petrochemical, shipping, transportation, municipal water-supply, recreational, and coastal-maritime influences. Lake Houston, Lake Livingston, Lake Conroe, and Clear Lake therefore provide a practical set of reservoirs for a concise technical baseline. The objective of this study was to describe reservoir-specific occurrence patterns of arsenic (As), cadmium (Cd), copper (Cu), lead (Pb), nickel (Ni), and zinc (Zn) and to interpret the results conservatively in relation to quality assurance/quality control (QA/QC) performance, regional context, and relevant drinking-water benchmarks.

## Materials and Methods

**Fig. 1:**
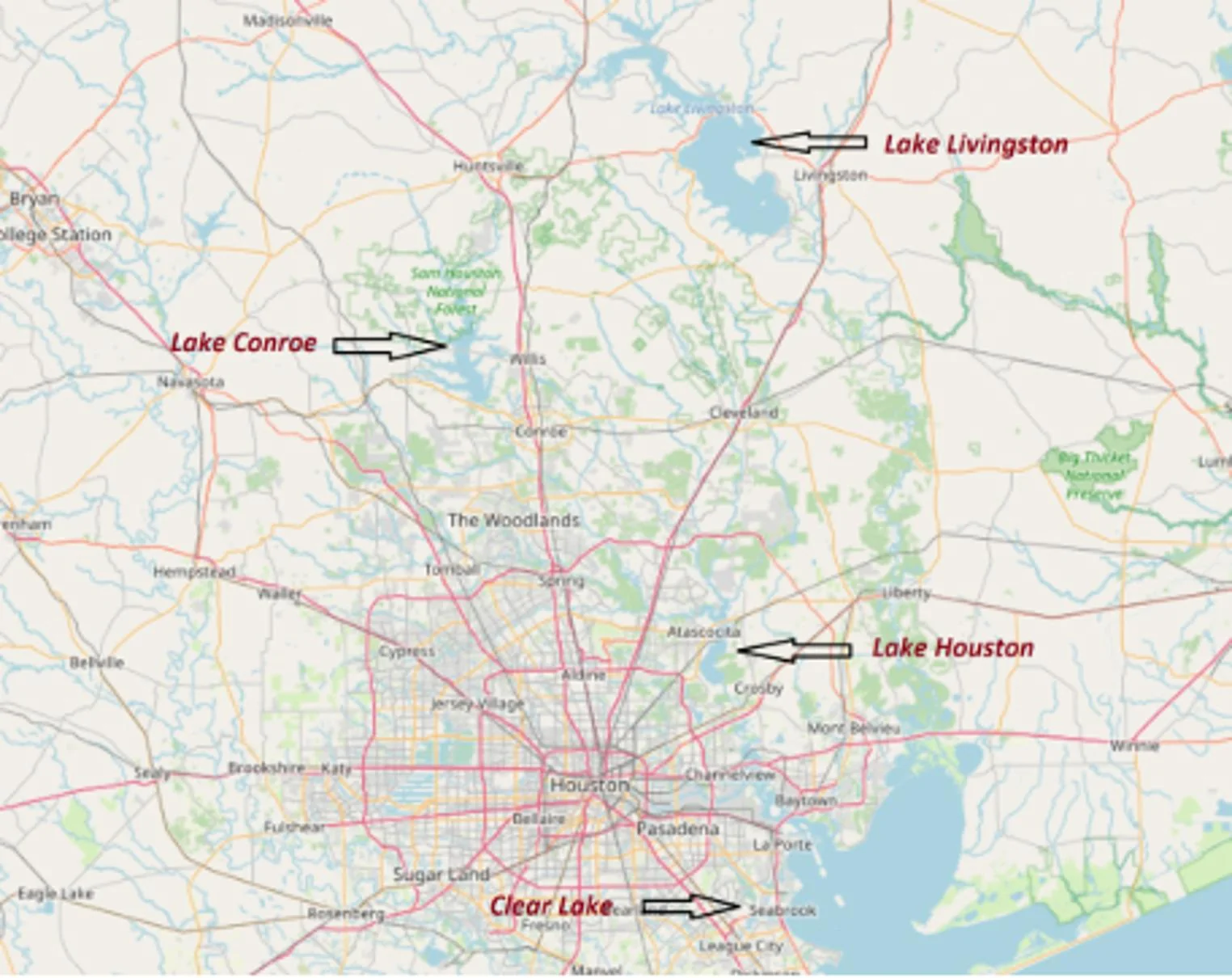
Approximate locations of Lake Houston, Lake Livingston, Lake Conroe, and Clear Lake in the greater Houston region. Base map adapted from OpenStreetMap

Surface-water samples were collected on January 28, 2018, from shoreline locations at Lake Houston, Lake Livingston, Lake Conroe, and Clear Lake (Fig. 1). Sampling locations were selected based on shoreline accessibility rather than proximity to drinking-water intake facilities. Three surface-water samples and one backup sample were collected at a single shoreline location per reservoir, with approximately 125 mL of water collected per sample in amber bottles. Samples were collected at approximately 20 cm below the water surface, placed on ice, filtered, and acidified with nitric acid for trace metal analysis. Target analytes included As, Cd, Cu, Pb, Ni, and Zn.

Trace metals were measured by inductively coupled plasma-mass spectrometry (ICP-MS) using an Agilent 7800 instrument following U.S. Environmental Protection Agency (U.S. EPA) Method 200.8 (U.S. EPA 1994). Concentrations are reported as µg L⁻¹, which is numerically equivalent to parts per billion in water. Instrumental limits of detection (LODs) were 0.05 µg L⁻¹ for As, 0.0043 µg L⁻¹ for Cd, 0.10 µg L⁻¹ for Cu, 0.02 µg L⁻¹ for Pb, 0.05 µg L⁻¹ for Ni, and 0.50 µg L⁻¹ for Zn.

QA/QC procedures included method blanks, calibration verification, matrix spikes, and certified standard reference materials. LODs and limits of quantitation were determined from seven replicate blank measurements. Mean recovery efficiencies for fortified samples ranged from 92% to 106%, and calibration curves had R² values greater than 0.999. Because the dataset represents one sampling period with limited replication, the analysis was treated as descriptive rather than inferential. Means and relative standard deviations (RSDs) were calculated from the analytical tables; zinc results below detection were retained as below detection limit (BDL) rather than estimated as positive concentrations.

## Results and Discussion

Trace metals were detected at low concentrations across the four reservoirs (Table 1). Copper was the dominant analyte in every reservoir, with the highest mean concentration in Lake Houston (15.5 µg L⁻¹), followed by Lake Conroe (14.2 µg L⁻¹), Lake Livingston (12.7 µg L⁻¹), and Clear Lake (9.82 µg L⁻¹). Low RSD values for Cu (2.16–2.64%) indicate relatively low variability in the reported measurements and identify Cu as the clearest priority analyte for future monitoring.

**Table 1:** Mean trace metal concentrations in surface water from four Houston-area reservoirs

Metal	Lake Houston	Lake Livingston	Lake Conroe	Clear Lake	Range
As	1.71 (4.92)	1.50 (4.89)	1.46 (5.74)	1.58 (3.53)	1.46–1.71
Cd	0.049 (18.85)	0.010 (72.38)	BDL	0.016 (42.94)	BDL–0.049
Cu	15.5 (2.16)	12.7 (2.44)	14.2 (2.41)	9.82 (2.64)	9.82–15.5
Pb	0.770 (6.12)	0.320 (5.65)	0.182 (4.02)	0.248 (2.17)	0.182–0.770
Ni	1.23 (3.53)	1.95 (2.93)	0.331 (6.32)	0.936 (5.33)	0.331–1.95
Zn	2.62 (33.64)	BDL	BDL	1.04 (38.05)	BDL–2.62

Values are μg L⁻¹ with RSD (%) in parentheses. BDL = below detection limit

Arsenic was comparatively stable, ranging from 1.46 µg L⁻¹ in Lake Conroe to 1.71 µg L⁻¹ in Lake Houston. These values were far below the 10 µg L⁻¹ federal drinking-water maximum contaminant level (MCL) for arsenic (U.S. EPA 2026a). In Texas, low-level As occurrence should also be interpreted in relation to regional geogenic background in groundwater and water-supply systems (Reedy and Scanlon 2024). Lead concentrations were also low (0.182–0.770 µg L⁻¹) and remained below the 15 µg L⁻¹ drinking-water action level (U.S. EPA 2026b).

Cadmium was the least abundant analyte, ranging from below the detection limit to 0.049 µg L⁻¹. The relatively high RSD values for Cd in Lake Livingston and Clear Lake are consistent with greater analytical uncertainty at low concentrations. Nickel showed a different reservoir pattern from the other analytes, with the highest mean concentration in Lake Livingston (1.95 µg L⁻¹). This finding indicates that no single reservoir dominated all metals and that spatial ranking depends on the analyte considered.

Zinc was quantified in Lake Houston (2.62 µg L⁻¹) and Clear Lake (1.04 µg L⁻¹), whereas Lake Livingston and Lake Conroe were reported as BDL (< 0.50 µg L⁻¹). Retaining these BDL designations is important because Zn is often interpreted as a possible indicator of urban runoff, infrastructure, or built-environment inputs. Replacing BDL results with estimated positive concentrations would overstate the reservoir-wide Zn signal and weaken the baseline interpretation.

The present findings provide additional context for previous water-quality investigations in the Houston region. Van Metre and Sneck-Fahrer (2002) documented historical trace metal trends in Lake Houston sediments, while Adams (2025) examined spatial and temporal water-quality patterns in Lake Conroe. The relatively low As and Pb concentrations observed in this study can also be considered alongside regional drinking-water monitoring information (Houston Public Works 2024). However, differences in sampling media, locations, and water treatment limit direct quantitative comparisons with these previous investigations and drinking-water reports.

The main shortcoming of this technical note is its screening design: samples were collected during one sampling period with limited within-reservoir replication. Therefore, the results should not be used for formal source attribution, seasonal inference, or regulatory compliance conclusions. Their significance lies in establishing a concise QA/QC-supported baseline showing low-level trace metal occurrence, Cu dominance, and reservoir-specific patterns in a region where urban and geogenic influences can overlap (TCEQ 2024; Das et al. 2026). Future work should include seasonal sampling, storm-event sampling, additional within-reservoir locations, and paired measurements of water-quality variables to test whether Cu, Ni, Zn, or As patterns persist over time.

## Data Availability

The datasets generated and/or analyzed during the current study are available from the corresponding author upon reasonable request.
